# Novel validation of HDR brachy therapy dosimetry for cervical cancer using egs_brachy Monte Carlo simulations: a comparative analysis with Oncentra treatment planning system

**DOI:** 10.1002/acm2.70144

**Published:** 2025-07-13

**Authors:** Duong Thanh Tai, Nguyen Thi Anh Thu, Tran Thien Thanh, Pham Anh Tuan, Marc J. P. Chamberland, Peter Sandwall, David Bradley, James C. L. Chow

**Affiliations:** ^1^ Department of Medical Physics Faculty of Medicine Nguyen Tat Thanh University Ho Chi Minh City Vietnam; ^2^ Department of Nuclear Physics Faculty of Physics and Engineering Physics University of Science Ho Chi Minh City Vietnam; ^3^ Vietnam National University Ho Chi Minh City Vietnam; ^4^ Nuclear Medicine and Oncology Centre Bach Mai Hospital Ha Noi Vietnam; ^5^ Department of Radiation Oncology University of Vermont Medical Center Vermont USA; ^6^ Department of Radiation Oncology OhioHealth Mansfield Ohio USA; ^7^ Applied Physics and Radiation Technologies group CCDCU School of Engineering and Technology Sunway University Sunway City Malaysia; ^8^ School of Mathematics and Physics University of Surrey Guildford UK; ^9^ Department of Radiation Oncology University of Toronto Toronto Ontario Canada; ^10^ Radiation Medicine Program Princess Margaret Cancer Centre University Health Network Toronto Ontario Canada

**Keywords:** brachytherapy, dose calculations, egs_brachy, heterogeneity, Monte Carlo simulation, radiation treatment planning, TG‐186, TG‐43

## Abstract

**Purpose:**

This study aims to validate HDR brachytherapy dosimetry for cervical cancer patients utilizing the egs_brachy Monte Carlo (MC) simulation.

**Methods:**

Three cervical cancer patients treated with ^192^Ir HDR brachytherapy were included. Dose distributions were calculated by the Oncentra Brachy v4 treatment planning system (TPS) based on AAPM TG‐43. The newly developed eb_gui, an egs_brachy graphical user interface for MC simulations, was applied in recalculating dose distributions for 12 fractions using digital imaging and communications in medicine‐radiotherapy (DICOM‐RT) anatomical information. Comparisons were made for clinical target volume (CTV), bladder, and rectum using dose–volume histograms (DVH) and clinically relevant plan quality indices.

**Results:**

TPS‐calculated doses were greater than those obtained from MC simulations. For the CTV, the median percentage differences were 7.9% (Q1: 6.4%, Q3: 9.8%; range: 0.4%–10.4%) for D_90_. For the bladder, the median percentage differences were 0.7% (Q1: 0.4%, Q3: 2.3%; range: −9.4–5.4%) for D_2cc_. For the rectum, the median percentage differences were 3.6% (Q1: 2.8%, Q3: 5.6%; range: 0.9%–6.4%) for D_2cc_.

**Conclusion:**

CTV and critical organ doses calculated by the TPS were consistently greater than those obtained from MC simulations. This suggests that the TPS may overestimate dose distributions, especially in heterogeneous regions like the pelvis. These results emphasize the need for continued validation of TPS algorithms in HDR brachytherapy for cervical cancer.

## 1 INTRODUCTION

Cervical cancer remains a significant health concern worldwide, particularly in regions where access to preventive healthcare services may be limited, one instance being Vietnam, a populous middle‐income country, where cervical cancer ranks as the second most common gynecologic cancer, with more than 4000 new cases reported in 2020.^1^ It is commonly managed using various radiotherapy modalities, including external beam radiotherapy (EBRT) and high‐dose‐rate (HDR) brachytherapy.^2^, ^3^

Brachytherapy, a cornerstone of cervical cancer treatment,^4^ offers tailored radioactive source placement, dose rate, and treatment duration, specific to the tumor type and location, and individual patient characteristics. This modality not only serves as the standard treatment for cervical cancer but is also extensively used for other cancers such as prostate, breast, skin, and head and neck cancers. Over the years, advancements in brachytherapy have markedly evolved, leading to enhanced outcomes and reduced treatment‐related morbidities.^5^, ^6^

The accurate delivery of radiation doses in brachytherapy poses challenges,^6^, ^7^, ^8^ particularly in heterogeneous anatomical environments such as the pelvis. Traditionally, brachytherapy dose calculations have relied on the American Association of Physicists in Medicine (AAPM) Task Group 43 (TG‐43) formalism,^9^, ^10^ which assumes a homogeneous aqueous medium and neglects tissue heterogeneity effects and interparticle attenuation. Consequently, it does not account for heterogeneities internally or externally to the patient, potentially impacting upon the accuracy of dose calculations in regions close to air or bone.

Although the TG‐43 formalism has been extensively adopted in Treatment Planning Systems (TPS), its limitations in predicting dose distributions accurately, particularly in anatomically complex regions such as the cervix, are widely acknowledged.^11^, ^12^ To overcome these limitations and enhance treatment precision, the adoption of model‐based dose calculation algorithms (MBDCA) has increased.^11^, ^13^ This shift was catalyzed by inconsistencies in dose distributions noted with the traditional TG‐43 protocol, prompting recommendations in the AAPM TG‐186 report.^12^ This report advocates for the use of patient‐specific models with realistic tissue compositions over conventional water‐based models. Monte Carlo (MC) model‐based dose calculation algorithms have thus gained favor for their improved dosimetric accuracy.^14^, ^15^, ^16^, ^17^ MC simulations provide a detailed representation of radiation transport in tissues by simulating the interactions of radiation particles with matter at a microscopic level, thereby enhancing dose accuracy by accounting for tissue heterogeneity, scatter, and attenuation effects. MC simulations have been extensively explored for dose calculations in brachytherapy with notable contributions in HDR brachytherapy for cervical cancer.^18^, ^19^, ^20^ These studies demonstrate the potential of MC modeling in addressing limitations of traditional TG‐43 formalism, particularly in heterogeneous environments.

Several MC codes, including MCNP, TOPAS, and EGSnrc (egs_brachy), are currently utilized in brachytherapy dosimetry, with numerous studies conducting comparative analyses of dose calculations between these MC codes and traditional TPS. For instance, Peppa et al.^21^ conducted a dosimetric and radiobiological comparison of TG‐43 and MC calculations in ^192^Ir breast brachytherapy applications using the MCNP v.6.1 code. Their results indicated statistically significant overestimation of target DVH parameters by TG‐43, albeit small (on average, less than 2% for target coverage indices and 4% for homogeneity indices). Furthermore, Krstic et al.^16^ compared MCNP with a planning system in brachytherapy for cervical cancer. Their study found good agreement between phantoms, as well as between MCNP and the planning system. Additionally, McKeown^22^ investigated an MC model‐based dose calculation for ^103^Pd low‐dose rate treatments in 80 breast brachytherapy cases and ^125^I low‐dose rate in 76 prostate brachytherapy cases. They concluded that there was a slight overestimation of dose in target volumes and prostate organs‐at‐risk (OAR), and a potentially clinically significant underestimation of dose in the skin when using standard TG‐43 calculations compared to the more accurate model‐based dose calculation algorithms (MBDCA). Moreover, Thibodeau^23^ conducted radiobiological modeling of ^103^Pd permanent breast seed implant brachytherapy using MC dose calculations for three patients. Their results showed that the dose absorbed by the healthy tissues according to water‐based models was less than those predicted by their TG‐186 counterparts, particularly in the skin, ribs, heart, and lungs.

Building on these findings, this study aims to validate the HDR brachytherapy dose distributions calculated by an Oncentra Brachy TPS (Nucletron, an Elekta company, Elekta AB, Stockholm, Sweden) for cervical cancer patients against those obtained from egs_brachy MC simulations, highlighting the potential for enhancing treatment precision and outcomes.

This study stands out as novel and topical for several reasons. First, it specifically addresses the dosimetric challenges of HDR brachytherapy in cervical cancer, a clinically significant and anatomically complex region that has been underexplored in prior research. Although previous studies have largely focused on other anatomical sites such as the prostate and breast, this work emphasizes the importance of accurate dose calculations in cervical cancer treatment, where tissue heterogeneity plays a critical role. Moreover, by utilizing the EGSnrc‐based egs_brachy MC code in combination with the recently developed eb_gui interface, this study introduces a more advanced method for modeling tissue heterogeneity compared to traditional TG‐43 formalism. This novel approach provides greater precision in validating treatment planning systems (TPS), specifically Oncentra Brachy, offering insights that could significantly improve treatment accuracy and outcomes in HDR brachytherapy for cervical cancer patients.

## 2 MATERIALS AND METHODS

**FIGURE 1:**
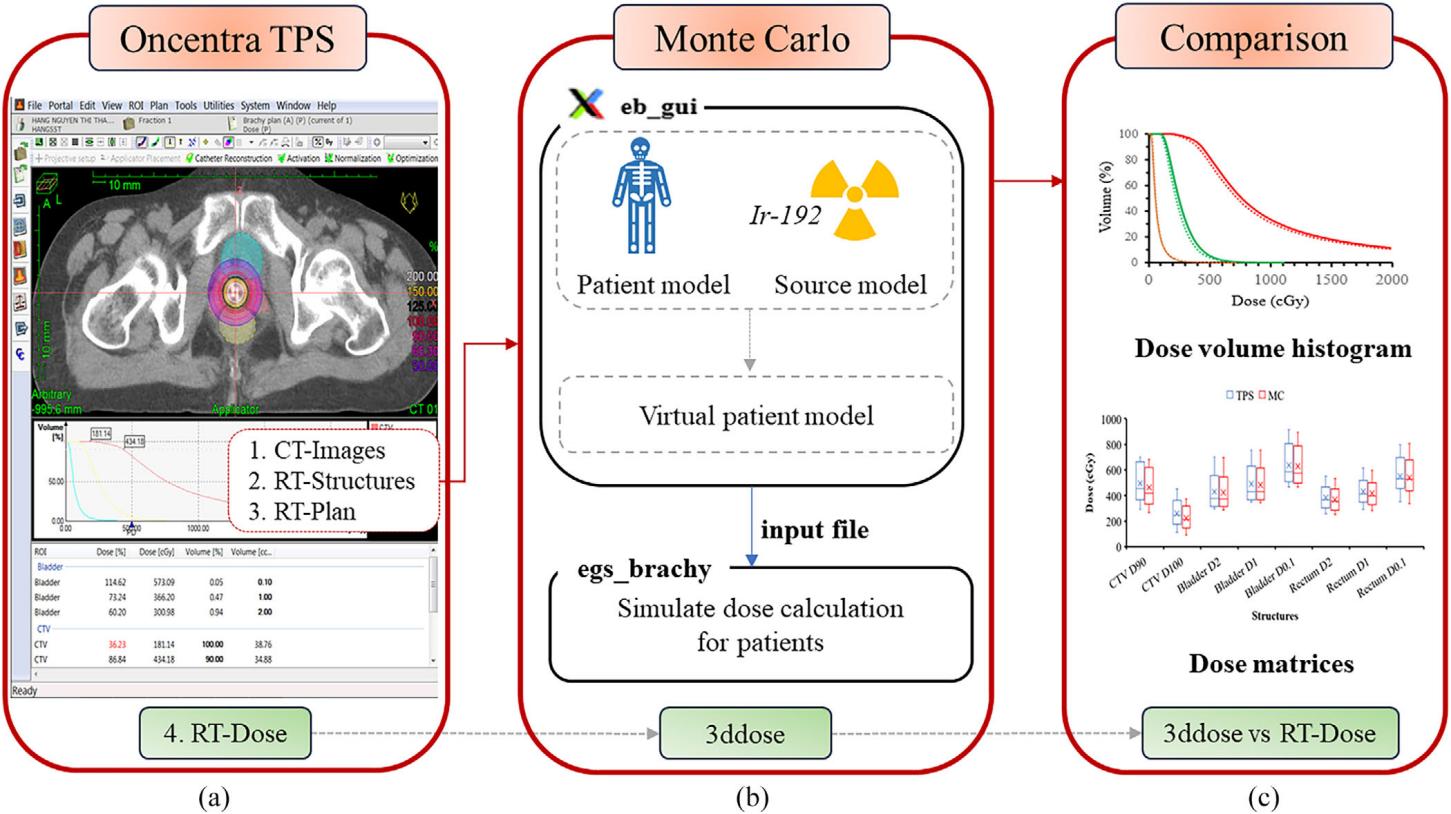
Flowchart showing the main process for comparing the MC and TPS: (a) a 3D dose distribution is calculated in the Oncentra TPS and DICOM RT files are exported; (b) the eb_gui application generates virtual patient models and input files for MC simulations with egs_brachy, and egs_brachy calculates 3D dose distributions; and (c) the TPS and MC dose distributions are compared. DICOM RT, digital imaging and communications in medicine radiotherapy; MC, Monte Carlo; TPS, treatment planning systems.

Figure 1 illustrates the main steps involved in comparing the MC simulations and the TPS calculations. Digital imaging and communications in medicine (DICOM) RT files are exported from the TPS. They are used to generate virtual patient models and input files for MC simulations. The TPS and MC dose distributions are then compared and analyzed.

As has been mentioned, this study involved three cervical cancer cases treated with high‐dose‐rate (HDR) brachytherapy at our institution. The prescribed dose per fraction and the total dose are presented in Table 4. The planning aim was to achieve optimal target coverage (CTV) while adhering to dose constraints for organs‐at‐risk (OARs), specifically the bladder and rectum, in alignment with GEC‐ESTRO recommendations.^24^

### 2.1 Treatment planning system

In this study, TPS dose calculations were carried out using Oncentra Brachy v4.3 software (Nucletron, an Elekta company, Elekta AB, Stockholm, Sweden), this employing a dose calculation algorithm based on the TG‐43 formalism. All treatment plans utilized the Utrecht Interstitial CT/MR Applicator Set (Elekta, Stockholm, Sweden), a versatile tool specifically designed for both interstitial and intracavitary gynecological brachytherapy.^25^ For each patient, high‐resolution computed tomography (CT) scans were imported into the Oncentra Brachy TPS, with a slice thickness of 3 mm and a matrix size of 512 × 512 pixels, and a voxel size of 0.6 mm × 0.6 mm × 3 mm in the *x*, *y*, and *z* directions, respectively. Within the TPS, individualized brachytherapy plans were meticulously designed for each patient. These plans included the specification of prescription doses, precise source (microSelectronHDR v2, Ir‐192) locations, and dwell times, optimizing the radiation dose distribution to the tumor while minimizing exposure to adjacent healthy tissues. Following the completion of the treatment planning, the radiation therapy (RT) plan, along with the associated RT structure and RT dose, was exported from the Oncentra Brachy system in DICOM format. These exports served as the foundational data sets for subsequent MC simulations, ensuring that the simulated environments closely replicated clinical conditions.

### 2.2 Monte Carlo simulation

CT images, RT plans, and RT structures exported in Section 2.1 were input into eb_gui^26^ to generate the necessary input files for egs_brachy simulations.^27^ Table 1 summarizes the MC simulation parameters.

**TABLE 1:** Summary of the MC simulation parameters.

Parameter	Description	Reference
Code	EGSnrc (CLRP fork, commit c4beb16) egs_brachy (commit 0219903)	[28, 29, 30]
Validation	EGSnrc/ egs_brachy validation	[30, 31, 32, 33, 34]
Timing	Approximately 4 h on AMD Ryzen 9 5900X (12 cores)	
Source	microSelectronHDR v2, Ir‐192	[31]
Simulation parameters	PCUT = 0.001 MeVECUT = 1.5 MeVTracklength scoringAll EGSnrc default parameters except: NRC bremsstrahlung cross sections Rayleigh scattering and electron impact ionization enabled	
Cross‐sections	XCOM database (AE = 0.512 MeV, AP = 0.001 MeV) Mass‐energy absorption coefficients calculated using the EGSnrc application “g”	
Histories (statistical uncertainty with parameter k = 1)	1 × 108 histories (< 2% on doses greater than 20% of the prescribed dose)	
DICOM CT Data	170–235 slices of 512 × 512 resolution	
Phantom	Type: EGS_XYZGeometry; Number of regions: 61603840; Average voxel volume: 0.00073985 cm^3^.	

Abbreviations: DICOM, digital imaging and communications in medicine; MC, Monte Carlo.

### 2.3 Tissue assignment schemes

Virtual patient models are constructed using recommendations from TG‐186.^12^ First, the mass density of each voxel is calculated using the CT calibration curve generated from the data in Table 2. Then, tissue type is assigned based on voxel mass density and location within or without a given structure. For example, all voxels within the bladder contour will be assigned bladder tissue type, with a density derived from the Hounsfield Unit (HU) value of the voxel. A voxel that does not belong to any contour will be assigned to be air if its mass density is below 0.6042 g/cm^3^, mean female soft tissue if its density is between 0.6042 g/cm^3^ and 1.10 g/cm^3^, and cortical bone if its mass density is greater than 1.10  g/cm^3^. Tissue assignment schemes^23^ used are summarized in Table 3.

**TABLE 2:** CT calibration curve for converting HU into mass densities for each voxel in the virtual patient model.

Hounsfield Units (HU)	Mass density (g/cm^3^)
−3025	0.001
−1000	0.001
0	1.008
61.9	1.073
1000	1.667
2000	2.3
3000	2.933
3100	2.999
5000	2.999
10000	7.365
20000	10
25000	10

Abbreviations: CT, computed tomography; HU, Hounsfield units.

**TABLE 3:** Tissue assignment schemes.

Structure	Tissue	Mass density range (in g/cm^3^)
Bladder	Bladder [35]	All
Rectum	Rectum [35]	All
All other voxels	Air [10]	< 0.6042
	Mean female soft tissue [36]	0.6042–1.10
	Cortical bone [35]	> 1.10

### 2.4 Metal artifact reduction

In order to create realistic models for simulating treatments, we have to consider the presence of the source within the simulation process. High‐density materials enveloping the source create the presence of high‐density voxels as well as streaking artifacts that can be seen throughout the virtual patient model. If not accounted for, the presence of these artifacts can cause tissue to be incorrectly assigned to those voxels, which can significantly affect dose distribution. Eb_gui uses the simple threshold replacement (STR) method described in Miksys et al.^34^ The user selects lower and upper limits of the density threshold to be applied in metal artifact reduction (MAR). MAR can be limited to certain contours if needed. For this work, we used the default density threshold, with any voxel within the target found to be outside of the upper threshold of 1.25 g/cm^3^ and the lower threshold of 0.75 g/cm^3^ being replaced with the mean density of the tissues forming the target of 1 g/cm^3^.

### 2.5 Absolute dose calculation

Calculated MC doses are reported as the dose‐to‐medium (D_m,m_), in units of gray per effective history. Doses are converted to units of gray using the approach described in the egs_brachy manual.^27^ All planning parameters used in the simulation and TPS are shown in Table 4, including the treatment fraction, prescription dose, air kerma strength, dwell time, and dose scaling factor (for the purpose of conversion from MC doses to absolute doses).

**TABLE 4:** Treatment parameters for HDR brachytherapy and dose scaling factor.

Patients	Fx	Dx (cGy)	Sk ($\mathrm{Gy}\;cm^{2}/h$)	$\Delta t_{\max}$ (s)	∑Tx (s)	F
A	1	650	404.8	25.2	316.8	2.44E+13
	2	650	379.1	25.7	297.4	2.33E+13
	3	650	355.0	12.7	177.8	1.08E+13
	4	650	332.5	31.6	260.2	2.52E+13
B	1	500	401.0	30.5	176.9	2.93E+13
	2	500	382.8	27.8	211.2	2.55E+13
	3	500	375.6	30.0	131.8	2.70E+13
	4	500	357.8	23.4	171.1	2.01E+13
C	1	750	351.8	35.4	355.6	2.98E+13
	2	750	326.2	27.0	486.0	2.11E+13
	3	750	308.6	70.3	408.5	5.19E+13
	4	750	288.8	90.0	581.6	6.22E+13

Abbreviations: Fx, fractions; Dx, prescription dose; HDR, high dose rate; Sk, air kerma strength; $\Delta t_{max},$ max dwell time; ∑Tx: Total treatment time; F, dose scaling factor.

### 2.6 Comparison of MC with TPS

To compare dose distributions, calculations from the Oncentra Brachy TPS using the TG‐43 formalism were juxtaposed with those derived from egs_brachy MC simulations. We examined the differences between the TPS and MC simulation using the D_90_ parameter for the target, as well as the D_2cc_ for the bladder and rectum. The D_90_ represents the minimum dose received by 90% of the target volume, while the D_2cc_ indicates the minimum dose received by the most exposed 2 cm^3^ of the organ at risk, such as the bladder or rectum. Percentage differences were computed using the following Equation 1.

(1) $$\mathrm{Difference}\;\left(\%\right)=\frac{\mathrm{TPS}\;\mathrm{dose}-\mathrm{MC}\;\mathrm{dose}}{\mathrm{MC}\;\mathrm{dose}}\;\times 100$$

Moreover, DVHs for CTV, rectum, and bladder were analyzed between the TPS and MC simulations. Dose metrics for CTV, bladder, and rectum across 12 fractions of treatment were also compared between the two methods.

## 3 RESULTS

Table 5 shows the computed D_90_ dose values at the CTV obtained through both TPS and MC simulation. Patients A, B, and C are prescribed doses of 650, 500, and 750 cGy, respectively. In Table 5, the percentage disparities are shown between values obtained in the TPS utilizing the TG‐43 method and MC simulation, ranging from 0.4% to 10.4% across various fractions. For the CTV, the percentage differences in D_90_ between TPS and MC simulations showed a median of 7.9% (Q1: 6.4%, Q3: 9.8%), with a range of 0.4% to 10.4% across all fractions.

**TABLE 5:** The difference between TPS and MC for CTV for D_90_.

Patients	Fx	Dx (cGy)	TPS (%)	MC (%)	Difference (%)
A	1	650	105.2	98.7	6.6
	2	650	53.8	48.7	10.4
	3	650	47.3	42.9	10.4
	4	650	73.9	67.3	9.7
B	1	500	86.8	79.9	8.7
	2	500	83.0	77.8	6.7
	3	500	58.7	53.2	10.3
	4	500	82.3	76.1	8.1
C	1	750	81.6	79.2	3.0
	2	750	100.2	94.6	5.9
	3	750	98.2	97.8	0.4
	4	750	86.4	80.2	7.6
Median			82.7	78.5	7.9
**Q1**			70	63.8	6.4
**Q3**			89.7	83.8	9.8
Range (min–max)			47.3–105.2	42.9–98.7	0.4–10.4

*Note*: Q1: First quartile; Q2: Second quartile.

Abbreviations: CTV, clinical target volume; Dx, prescription dose; Fx, fractions; MC, Monte Carlo; TPS, treatment planning system.

Table 6 shows the D_2cc_ values of the bladder computed by both the TPS and MC simulation across all patients. The percentage variance of D_2cc_ ranges from −9.4% to 5.4%. For the bladder, the D_2cc_ percentage differences between TPS and MC simulations exhibited a median of 0.7% (Q1: 0.4%, Q3: 2.3%), with a range of −9.4% to 5.4% across all fractions.

**TABLE 6:** The difference between TPS and MC for bladder for D_2cc_.

Patients	Fx	Dx (cGy)	TPS (%)	MC (%)	Difference (%)
A	1	650	89.4	98.6	−9.4
	2	650	71.1	67.9	4.8
	3	650	50.1	48.4	3.4
	4	650	58.3	57.9	0.7
B	1	500	60.2	62.2	−3.3
	2	500	70.9	67.3	5.4
	3	500	58.8	57.8	1.9
	4	500	62.2	62.0	0.3
C	1	750	68.0	67.8	0.4
	2	750	86.9	86.2	0.8
	3	750	53.5	52.5	1.8
	4	750	100.0	99.6	0.4
Median			65.1	64.8	0.7
**Q1**			58.7	67.9	0.4
**Q3**			75.1	72.5	2.3
Range (min–max)			50.1–100.0	48.4–99.6	−9.4–5.4

Abbreviations: CTV, clinical target volume; Dx, prescription dose; Fx, fractions; MC, Monte Carlo; TPS, treatment planning system.

Similar to Table 6, Table 7 illustrates the D_2cc_ values for the rectum derived from both the TPS and MC simulation. Maintaining identical patient prescriptions (ensuring use of the same prescribed dose for all patients included in the study), the percentage discrepancy between the TPS and MC simulation ranged from 0.9% to 6.4%. For the rectum, the D_2cc_ percentage differences between TPS and MC simulations demonstrated a median of 3.6% (Q1: 2.8%, Q3: 5.6%), with a range of 0.9% to 6.4% across all fractions.

**TABLE 7:** The difference between TPS and MC for rectum for D_2cc._

Patients	Fx	Dx (cGy)	TPS (%)	MC (%)	Difference (%)
A	1	650	53.6	50.7	5.6
	2	650	55.5	53.8	3.1
	3	650	39.7	38.7	2.7
	4	650	41.4	39.2	5.5
B	1	500	89.8	84.4	6.4
	2	500	74.4	71.6	3.9
	3	500	60.8	57.2	6.4
	4	500	60.3	57.1	5.7
C	1	750	70.4	69.7	0.9
	2	750	73.5	71.1	3.3
	3	750	50.4	49.3	2.2
	4	750	63.2	61.4	2.8
Median			60.6	57.2	3.6
**Q1**			52.8	50.4	2.8
**Q3**			71.1	70.1	5.6
Range (min–max)			39.7–89.8	38.7–84.4	0.9–6.4

Abbreviations: CTV, clinical target volume; Dx, prescription dose; Fx, fractions; MC, Monte Carlo; TPS, treatment planning system.

**FIGURE 2:**
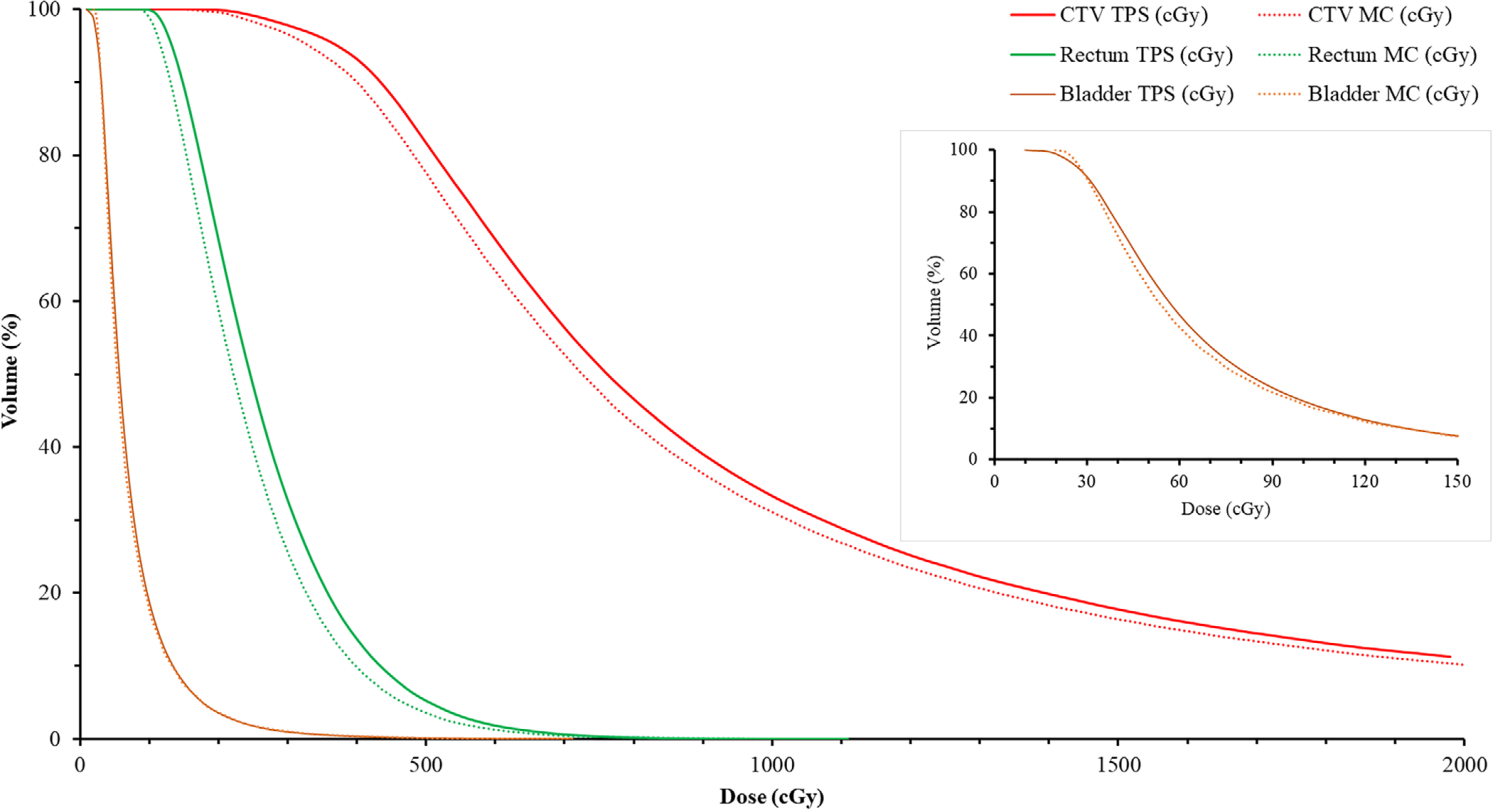
Comparison of DVHs between TPS and MC for Fraction #1 of Patient A. This figure shows the DVHs for the CTV, rectum, and bladder, comparing the TPS based on the TG‐43 formalism (solid lines) with MC simulations (dashed lines). The inset shows the bladder DVH on a dose scale of 0–150 cGy. CTV, clinical target volume; DVHs, dose–volume histograms; MC, Monte Carlo, TPS, treatment planning system.

**FIGURE 3:**
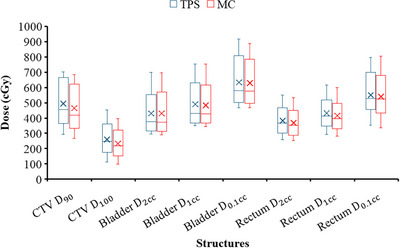
Boxplots comparing the dose metrics calculated by TPS and MC for a total of 12 fractions of brachytherapy for the three cervical cancer patients. The line within each of the boxplots represents the median value, while “x” indicates the mean value of the distribution. MC, Monte Carlo; TPS, treatment planning system.

Making use of the example first fraction treatment plan obtained for Patient A, Figure 2 illustrates the dose–volume relationships of the CTV, rectum, and bladder. Figure 3 presents boxplots showcasing the dose metrics of the aforementioned volumes‐of‐interest.

## 4 DISCUSSION

Although previous research has demonstrated the advantages of MC simulation for prostate and breast cancer brachytherapy,^13^, ^22^, ^23^ this study extends that validation to cervical cancer, itself presenting its own distinct dosimetric challenges due to the anatomical heterogeneity of the pelvic region. By utilizing a combination of egs_brachy and the newly developed eb_gui, we achieved more accurate modeling of tissue heterogeneity, setting this work apart from earlier studies.^14^, ^15^, ^37^ The eb_gui interface simplifies and enhances the precision of input preparation, enabling more efficient recalculation of clinical treatment plans. This approach marks a significant advancement in improving dose accuracy and, consequently, patient outcomes in HDR brachytherapy for cervical cancer.

For the D_90_ values of the CTV in brachytherapy, calculated using both the TPS and MC simulation, it is seen that noticeable variations are observed. For instance, in Patient A, the percentage differences between TPS and MC range from 6.6% to 10.4%, indicating significant disparities in dose calculation methods. Similar trends are observed for Patients B and C, albeit with varying magnitudes. Patient C exhibits relatively lower percentage differences, ranging from 0.4% to 7.6%, compared to Patients A and B. However, the median percentage difference across all three patients and fractions stands at 7.9% (range: 0.4%–10.4%). These findings suggest that while there is consistency in the trend of higher doses calculated by TPS compared to MC, the extent of this difference varies among patients and treatment fractions.

For the D_2cc_ values of the bladder in brachytherapy, highlighting the comparison between calculations made using the TPS and MC simulation, alongside their percentage differences. As the bladder is a critical organ that should be spared during treatment, analyzing these results is crucial in assessing treatment planning accuracy. Across different patients and fractions, varying degrees of difference between TPS and MC calculations are observed. For instance, in Patient A, the percentage differences range from −9.4% to 4.8%, indicating notable disparities in bladder dose estimations. Patient B exhibits a wider range of differences, with some fractions showing negative differences, implying higher doses calculated by TPS compared to MC, while others show positive differences. Conversely, Patient C demonstrates relatively minor differences, with percentages ranging from 0.0% to 1.7%. The median percentage difference across all patients and fractions is 0.7% (range: −9.4%–5.4%). These findings underscore the importance of accurate bladder dose estimation to minimize the risk of radiation‐induced toxicity and to ensure optimal treatment outcomes. Although overall differences are relatively small, individual patient variations emphasize the need for careful consideration of dose calculation methods to effectively spare critical organs like the bladder during brachytherapy. Similarly, Table 6 outlines the D_2cc_ values for the rectum in cervical brachytherapy. Given the critical nature of the rectum as another organ to be spared during treatment, these findings are essential for evaluating treatment planning accuracy. Patient A shows percentage differences ranging from 2.7% to 5.6%, indicating notable disparities in rectal dose estimations. Patient B exhibits a similar trend, with percentage differences ranging from 3.9% to 6.4%. Patient C demonstrates relatively smaller differences, with percentages ranging from 0.9% to 3.3%. However, the median percentage difference across all patients and fractions is 3.6% (range: 0.9%–6.4%). These results emphasize the importance of accurate rectal dose estimation to mitigate the risk of radiation‐induced toxicity and ensure optimal treatment outcomes in brachytherapy. Although the overall differences are moderate, individual patient variations underscore the necessity of carefully considering dose calculation methods to effectively spare critical organs like the rectum during treatment planning.

Based on the DVHs (Figure 3) of the CTV, bladder, and rectum obtained in this study, it is evident that there exists a consistent trend wherein DVH curves calculated by the TPS tend to exhibit larger doses for the same volumes compared to those computed by MC simulation. This discrepancy is indicative of the TPS potentially overestimating the dose distribution within these target volumes and critical organs. Such overestimation, particularly in the CTV, could imply a risk of delivering lower doses than intended, potentially leading to adverse treatment outcomes. Conversely, for organs‐at‐risk such as the bladder and rectum, the overestimated doses depicted by TPS may suggest a decreased risk of radiation‐induced toxicity in the real situation, predicted by the MC simulation. Therefore, careful consideration of these differences is paramount in treatment planning to ensure accurate dose delivery to the target while minimizing the risk of harm to critical organs. Further investigation into the underlying causes of these disparities would be crucial for refining treatment planning methodologies and optimizing patient outcomes in brachytherapy.

Based on the Boxplots (Figure 3), the boxplots reveal that the mean values (marked by the symbol “X” inside the boxes) of dose metrics from the TPS are consistently greater than those from the MC simulations across most structures and fractions. This systematic difference suggests that the TPS may overestimate doses compared to the MC simulations, which could be due to the assumption of tissue homogeneity in the TG‐43 formalism used by the TPS. The consistent overestimation of doses by the TPS could lead to conservative treatment planning, potentially reducing the effectiveness of the therapy due to suboptimal dosing. Conversely, underestimation might increase the risk of toxicity. These discrepancies underscore the importance of choosing the appropriate computational model for dose calculation in clinical practice and highlight the need for robust verification methods, such as MC simulations, to validate TPS results.

In summary, the MC doses are systematically lower than the TPS. This can be attributed to differences in mass energy absorption coefficients of the tissues for the energy range of ^192^Ir. The TG‐43 formalism employed by the TPS assumes a homogeneous water‐equivalent medium, which simplifies dose calculations but may not accurately reflect the heterogeneity of human tissues. Notably, the mass energy absorption coefficient for the bladder is more akin to that of water than that of other tissues such as the rectum and CTV. This distinction partially explains the less pronounced dose discrepancies in the bladder observed in our results. These findings underscore the importance of incorporating realistic tissue‐specific absorption properties in dose calculations to enhance the precision of HDR brachytherapy planning. In our study, we observed differences between TG‐43 and MC calculations exceeding the clinically acceptable threshold of 5% in several cases, particularly for D90 of the CTV and D2cc of the bladder and rectum. According to Kirisits et al.,^38^ such discrepancies warrant careful consideration due to their potential impact on clinical outcomes. The observed differences highlight the limitations of TG‐43 in accurately accounting for tissue heterogeneities and applicator material properties. To address these challenges, we recommend the integration of MC‐based verification methods as a secondary QA tool, alongside potential adaptations to treatment protocols when significant discrepancies are identified.

Several key studies^17^, ^18^, ^19^ demonstrate similar findings regarding the overestimation of dose distributions by TG‐43 when compared to MC simulations. These studies align with our results, further validating the observed differences between these two methods. Although TG‐43 remains the clinical standard, our results suggest that recalibrating clinical protocols to incorporate MC‐based dose calculations may lead to more accurate treatment planning and potentially improve patient outcomes. This is especially relevant for ensuring adequate target coverage while minimizing dose to critical organs. The clinical implications of our findings highlight the need to reevaluate the continued reliance on TG‐43 dose calculations, particularly in anatomically complex cases such as cervical cancer. Although TG‐43 provides a simplified framework, it has inherent limitations. For instance, as demonstrated in this study, differences exceeding ± 5% may necessitate adjustments to treatment protocols or prescription doses. However, as stated in TG‐186, societal recommendations for dose prescriptions remain tied to TG‐43 until sufficient clinical data can be obtained to justify a shift without equivocation. This underscores the importance of further research and collaboration within the brachytherapy community to establish robust guidelines for implementing model‐based dose calculation algorithms in routine clinical practice. Although the transition to model‐based dose calculation algorithms is not without challenges, including computational demands and the need for reoptimization workflows, it represents a critical step toward improving treatment accuracy and patient safety. Based on our findings, we strongly encourage clinics to urgently consider integrating MC‐based methods as a secondary verification tool for HDR brachytherapy plans. By disseminating findings like ours more broadly, we aim to contribute to the growing body of evidence supporting the integration of MBDCAs into clinical workflows. Future studies should focus on addressing the barriers to adoption, such as computational feasibility and clinical training, to accelerate the transition from TG‐43 to more advanced dosimetric techniques.

In addition, as the Utrecht applicator is constructed from plastic rather than metal, the potential dosimetric uncertainties associated with metal artifact reduction techniques are expected to be negligible. However, Miksys et al.^39^ demonstrated that differences between MAR and non‐MAR techniques can reach up to 6% for PTV D_90_ and 13% for skin D_1cc_ in certain scenarios. Future research should explore the dosimetric impact of applicators made from different materials, particularly metal‐based ones, to assess the role of MAR techniques and their influence on dose accuracy. Furthermore, electron transport was not performed (ECUT = 1.5 MeV) in this study due to the plastic composition of the Utrecht Interstitial CT/MR Applicator. However, for metal‐based applicators, where electron transport may significantly influence dose deposition,^40^, ^41^ future studies should investigate the role of enhanced electron scattering and its impact on tissue regions adjacent to metal applicators. Moreover, the current study serves as an initial exploration into the feasibility and clinical application of integrating MC‐based dose calculations using the egs_brachy framework. To draw statistically significant conclusions and generalize the findings to a broader population, future research should include a larger patient cohort. Expanding the sample size will enhance the robustness and clinical relevance of this approach.

Although MC simulations are widely regarded as the gold standard for dose calculations in brachytherapy, they are not immune to dosimetric uncertainties.^42^ These uncertainties stem from multiple sources, including limitations in source geometry modeling, voxel resolution, material composition, and statistical variance in particle histories. A recent comprehensive review by Adhikari et al. (2025)^43^ highlights that, while MC‐based dosimetry typically exhibits smaller standard deviations compared to experimental measurements, careful validation and quantification of both statistical (Type A) and systematic (Type B) uncertainties are critical to ensure clinical reliability. In accordance with the recommendations from AAPM Task Group 138 and the Groupe Européen de Curiethérapie–European Society for Therapeutic Radiology and Oncology (GEC‐ESTRO),^44^ uncertainty analysis in brachytherapy dosimetry should encompass both Type A uncertainties (evaluated using statistical methods) and Type B uncertainties (associated with modeling, measurement, or systematic limitations). For our egs_brachy simulations, Type A uncertainty was determined using 10⁸ particle histories per case, resulting in voxel‐level statistical uncertainties below 2% in regions receiving more than 20% of the prescribed dose—consistent with prior studies employing egs_brachy for HDR brachytherapy, such as Peppa et al.,^13^ Fletcher et al. 2023,^45^ and Moghadam et al.^46^ Additionally, we accounted for potential Type B uncertainties stemming from systematic effects, including CT‐to‐density conversion, source and applicator modeling, tissue segmentation, and voxel resolution. Although not individually quantified in this study, these sources are estimated to contribute an additional 3%–5% uncertainty, as suggested by the AAPM HEBD Report‐229,^47^ TG‐186,^12^ and TG‐268.^48^ Consequently, the combined Type A and Type B uncertainty budget of approximately 5% supports the reliability of the dose comparisons between MC simulations and the TPS presented in this work.

The novelty of this paper lies in its validation of HDR brachytherapy dosimetry for cervical cancer using the egs_brachy MC simulation with a newly developed graphical user interface, eb_gui. Unlike prior studies, this work directly compares dose distributions from the Oncentra TPS with MC recalculations based on patient‐specific anatomical information. The detailed analysis of DVH for CTV and critical organs highlights potential overestimations by the TPS, underscoring the need for more robust MC‐based dosimetric validation in HDR brachytherapy.

## 5 CONCLUSION

In conclusion, this study provides valuable insights into the dosimetric accuracy of HDR brachytherapy treatment planning for cervical cancer patients using the Oncentra Brachy TPS, validated against the egs_brachy MC simulation. Through comprehensive analysis of dose–volume metrics for tumor targets, and critical organs such as bladder and rectum, notable differences between TPS and MC calculations have been observed. Specifically, TPS consistently yields a higher dose for the same volume in the DVH compared to MC simulation, indicating a potential tendency for TPS to overestimate dose distributions. Although such discrepancies may pose risks of unintended underdosage to the CTV, they at the same time overestimated the radiation‐induced toxicity to organs‐at‐risk like the bladder and rectum. This underscores the critical importance of accurate dose estimation in treatment planning. Addressing these disparities through further refinement of dosimetric methodologies and ongoing validation efforts is essential to enhance treatment precision and optimize patient outcomes in HDR brachytherapy for cervical cancer.

## DISCLOSURES

All authors declare that they have nothing to disclose

## AUTHOR CONTRIBUTIONS

Duong Thanh Tai, Nguyen Thi Anh Thu, Marc J. P. Chamberland, and James C. L. Chow: Conceptualization, methodology, simulating and data colleting, writing – original draft; Duong Thanh Tai, Pham Anh Tuan, Tran Thien Thanh: Formal analysis, review & editing; Duong Thanh Tai, Tran Thien Thanh, Peter Sandwall, David Bradley, James C. L. Chow: revised and approved the final manuscript.

## CONFLICT OF INTEREST STATEMENT

The authors declare no conflicts of interest.

## Data Availability

Research data will be shared upon request to the corresponding author
